# Supplementary material for: Molecular basis of potent antiviral HLA-C-restricted CD8+ T cell response to an immunodominant SARS-CoV-2 nucleocapsid epitope
Source: Nat Commun. 2025 Aug 28;16:8062. doi: 10.1038/s41467-025-63288-3 (PMC12394707; doi:10.1038/s41467-025-63288-3)
Supplement: Supplementary file 1 — Supplementary Information [file 41467_2025_63288_MOESM1_ESM.pdf]

Molecular basis of potent antiviral HLA-C-restricted CD8+ T cell response to an immunodominant SARS-CoV-2 nucleocapsid epitope

Yoshihiko Goto[1, 2, #], You Min Ahn[3, 4, #], Mako Toyoda[1], Hiroshi Hamana[5], Yan Jin[1], Yoshiki Aritsu[1], Takeshi Nakama[1], Yuka Tajima[1,2], Janesha C. Maddumage[3, 4], Huanyu Li[1], Mizuki Kitamatsu[6], Hiroyuki Kishi[5], Akiko Yonekawa[7], Dhilshan Jayasinghe[3, 4], Nobuyuki Shimono[7], Yoji Nagasaki[8], Rumi Minami[9], Takashi Toya[10], Noritaka Sekiya[11,12], Yusuke Tomita[2], Demetra S.M. Chatzileontiadou[3, 4, 13], Hirotomo Nakata[14], So Nakagawa[15], Takuro Sakagami[2], Takamasa Ueno[1], Stephanie Gras[3, 4, 13*], Chihiro Motozono[1*]

[1] Division of Infection and immunity, Joint Research Center for Human Retrovirus infection, Kumamoto University, Kumamoto 8600811, Japan

[2] Department of Respiratory Medicine, Faculty of Life Sciences, Kumamoto University, Kumamoto 8608556, Japan

[3] Immunity and Infection program, La Trobe Institute for Molecular Science (LIMS), La Trobe University, Bundoora, VIC 3086, Australia.

[4] Department of Biochemistry and Chemistry, School of Agriculture, Biomedicine and Environment, La Trobe University, Bundoora, VIC 3086, Australia.

[5] Department of Immunology, Faculty of Medicine, Academic Assembly, University of Toyama, Toyama 9300194, Japan

[6] Department of Applied Chemistry, Faculty of Science and Engineering, Kindai University, Osaka 577-8502, Japan

[7] Center for the Study of Global Infection, Kyushu University Hospital, Kyushu University, Fukuoka 8128582, Japan

[8] Division of Infectious Diseases, Clinical Research Institute, National Hospitalization Organization, Kyushu Medical Center, Fukuoka 8108563, Japan

[9] Internal Medicine, Clinical Research Institute, National Hospital Organization, Kyushu Medical Center, Fukuoka 8108563, Japan

[10] Hematology Division, Tokyo Metropolitan Cancer and Infectious Diseases Center
Komagome Hospital, Tokyo 1138677, Japan.

[11] Department of Infection Prevention and Control, Tokyo Metropolitan Cancer and
Infectious Diseases Center Komagome Hospital, Tokyo 1138677, Japan.

[12] Department of Infectious Disease Emergency Preparedness, Institute of Science Tokyo, Tokyo 1138510, Japan.

[13] Department of Biochemistry and Molecular Biology, Monash University, Clayton, Victoria 3800, Australia.

[14] Department of Hematology, Rheumatology and Infectious Diseases, Kumamoto University School of Medicine, Kumamoto University Hospital, Kumamoto 8608556, Japan
[15] Department of Molecular Life Science, Tokai University School of Medicine, Isehara, Kanagawa 2591193, Japan

[#]These authors contributed equally
*Correspondence: S.Gras@latrobe.edu.au (S.G), motozono@kumamoto-u.ac.jp (C.M)

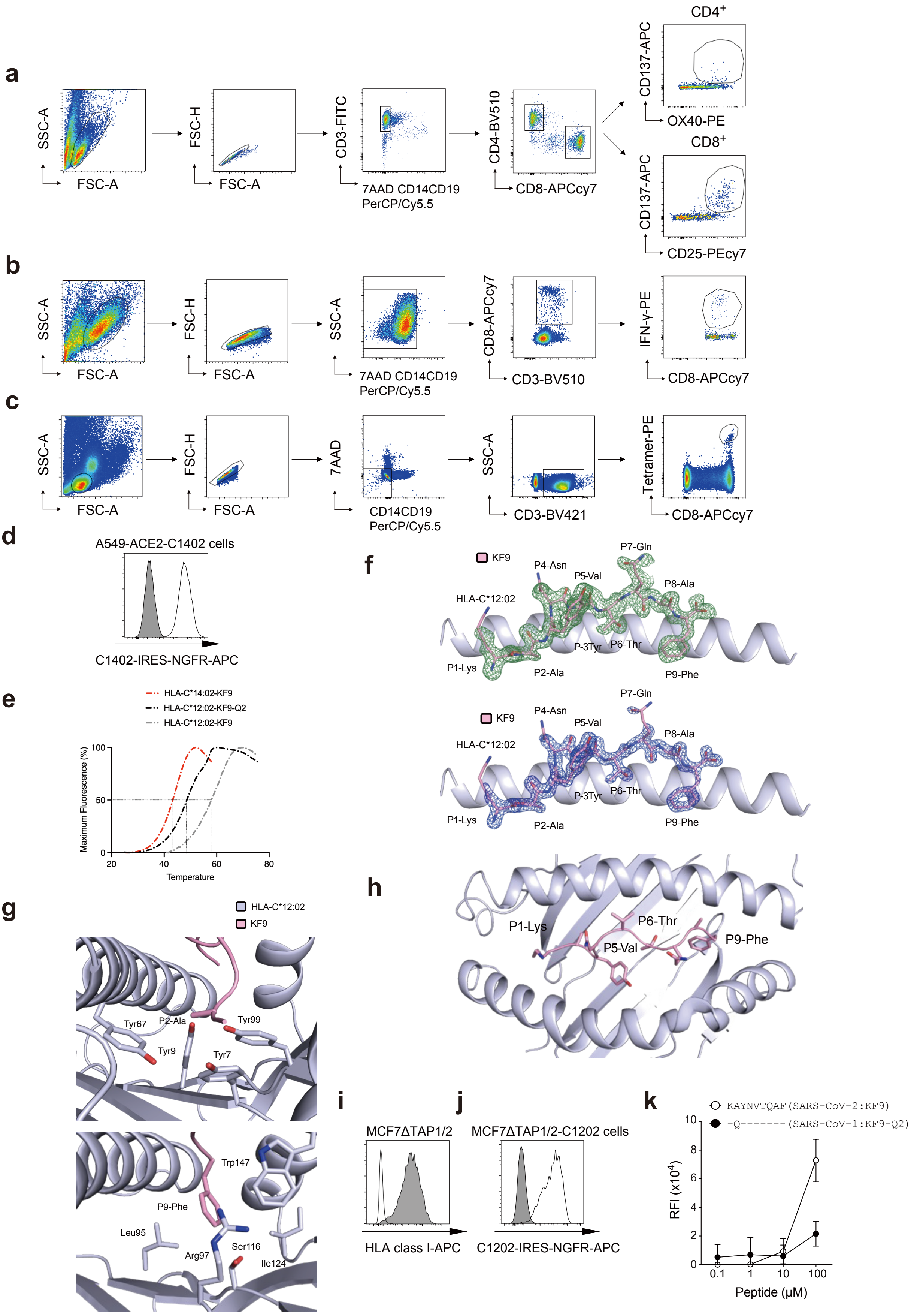

Supplementary Fig. 1 | Gating strategy and T cell and cell lines used in this study and crystal structure of HLA-C*12:02-KF9.
a-c Flow cytometry gating strategy of CD25^{+}CD137^{+} activated T cell lines from donor KK-006 (**a**), IFN-γ^{+} activated T cell lines (**b**), tetramer staining of PBMCs (**c**). **d** NGFR expression of A549-ACE2-C1402-IRES-NGFR cells. A549-ACE2 parental (shaded histogram) and FACS-sorted A549-ACE2-C1402-IRES-NGFR cells (open histogram) are shown. **e** Thermal melt curve (Tm) plots showing the normalised fluorescence intensity versus temperature for HLA-C*12:02-KF9 (grey line), HLA-C*12:02-KF9-Q2 (black line) and HLA-C*14:02-KF9 (red line) complexes at 5 μM. **f** Electron density map around the KF9 peptide (pale pink sticks) bound HLA-C*12:02 (blue cartoon), with the Fo-Fc map contoured in green at 3 σ and the 2Fo-Fc map contoured in blue at one σ. **g** The structure of HLA-C*12:02 (blue cartoon) showing the B pocket and F pocket residues surrounding the P2-Ala and P9-Phe of the KF9 peptide (pale pink sticks), respectively. **h** Top view of the HLA-C*12:02 (blue cartoon) presenting the KF9 peptide (pale pink sticks). **i** MCF7 cells were transfected with TAP1/2 CRISPER-Cas9 KO plasmid. MCF7 parental (shaded histogram) and HLA class-negative FACS-sorted MCF7ΔTAP1/2 (open histogram) are shown. **j** NGFR expression of MCF7ΔTAP1/2-C1202-IRES-NGFR cells. MCF7ΔTAP1/2 parental (shaded histogram) and MCF7-TAP1/2KO-C1202-IRES-NGFR cells (open histogram) are shown. **k** HLA expression is illustrated as relative median fluorescence intensity (RFI) as compared to unloaded MCF7-TAP1/2KO-C1202-IRES-NGFR cells at 26°C.

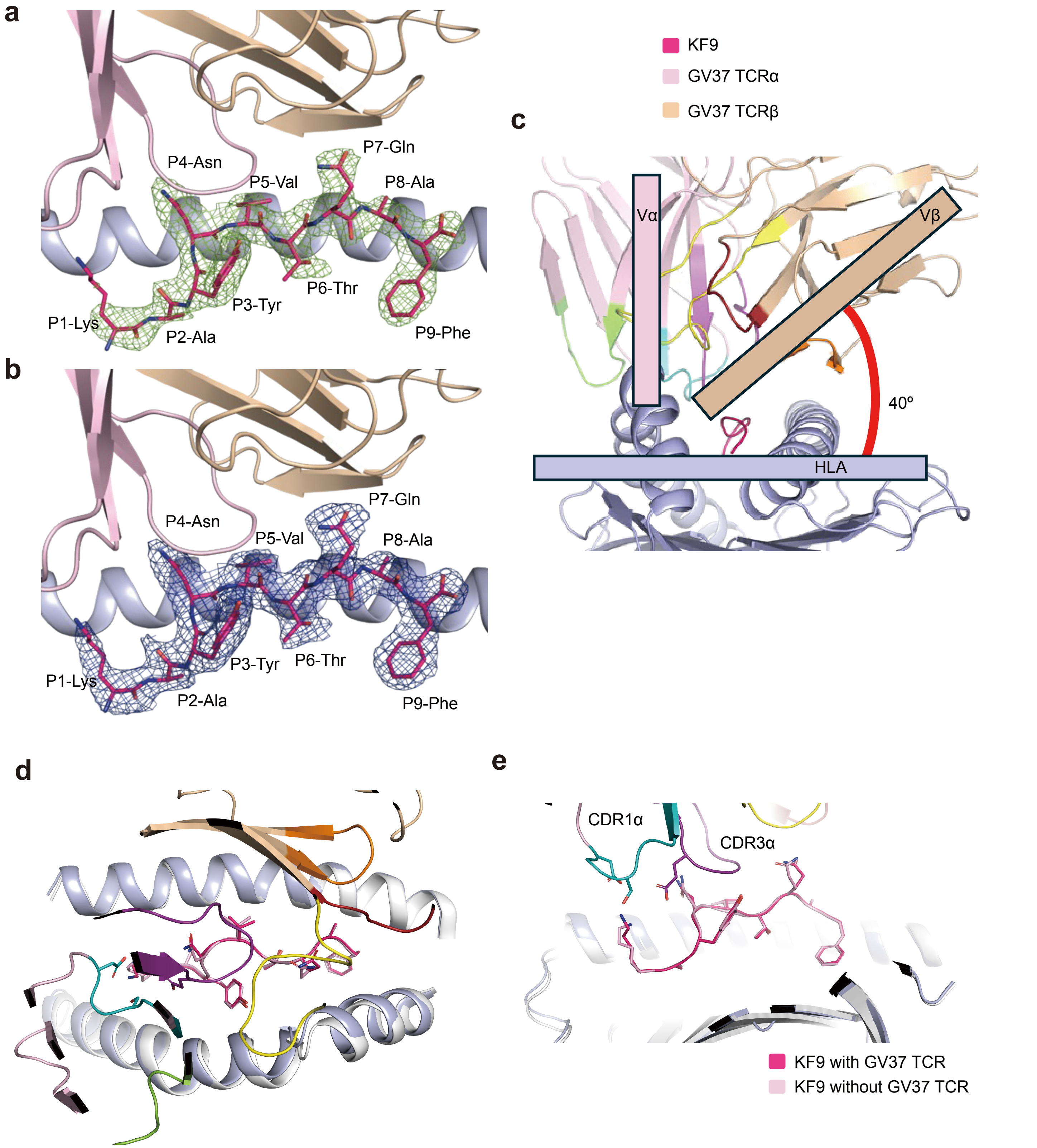

Supplementary Fig. 2 | Crystal structure of the GV37 TCR in complex with HLA-C*12:02-KF9.
a-b Electron density maps around the KF9 peptide (hot pink stick) presented by HLA-C*12:02 (blue cartoon) in complex with the GV37 TCR (TCRα chain in light pink and TCRβ chain in beige); (**a**) The Fo-Fc map (green) around the peptide is contoured at 3 σ, (**b**) The 2Fo-Fc map (blue) around the peptide is contoured at one σ. **c** The TCR Vβ chain (beige) binds to the HLA-C*12:02-KF9 complex with a 40° angle, while the Vα chain (light pink) interacts perpendicularly with the HLA-C*12:02-KF9. **d-e** Top and side views of the overlay of the crystal structures of HLA-C*12:02-KF9 with (HLA in blue cartoon and peptide in hot pink sticks) and without the GV37 TCR (HLA in white cartoon and peptide in light pink sticks).

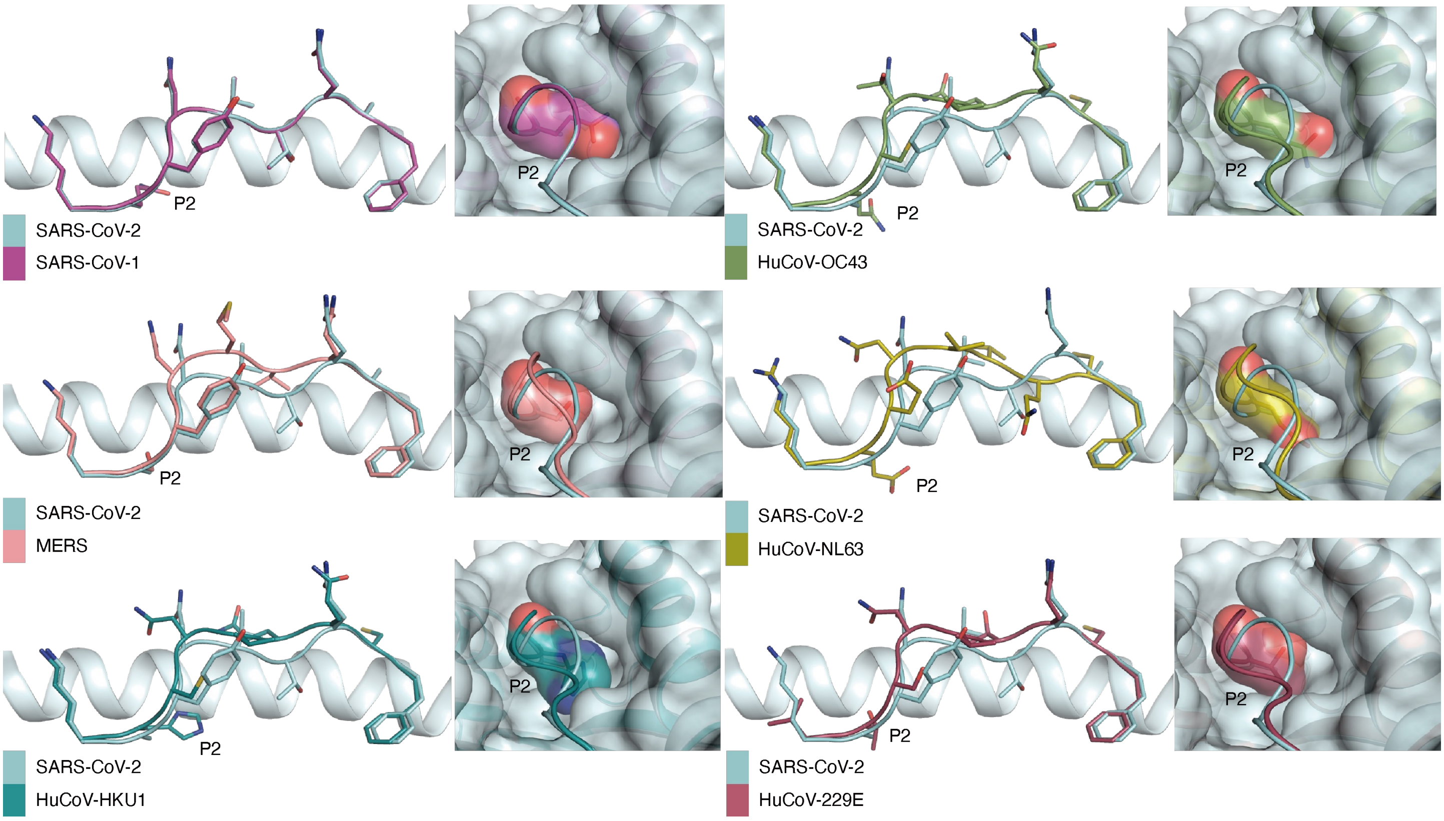

Supplementary Fig. 3 | Structural prediction of KF9 homologous peptides in complex with HLA-C*12:02 molecule.

The SARS-CoV-2-FK9 peptide is shown as a stick/cartoon representation in pale blue, similar to the HLA-C12:02 α1-helix. Each panel displays a superimposition of SARS-CoV-2-FK9 (blue) with SARS-CoV-1-FK9 (purple), MERS-KF9 (pink), HKU1-KF9 (blue), OC43-KF9 (green), NL63-KF9 (yellow), or 229E-KF9 (brown). Next to the peptide superimposition, a close-up of the B pocket of HLA-C12:02, represented as a blue surface, shows the P2 docking of each KF9 homologous peptide, with their P2 residues shown as surfaces in the corresponding color scheme.

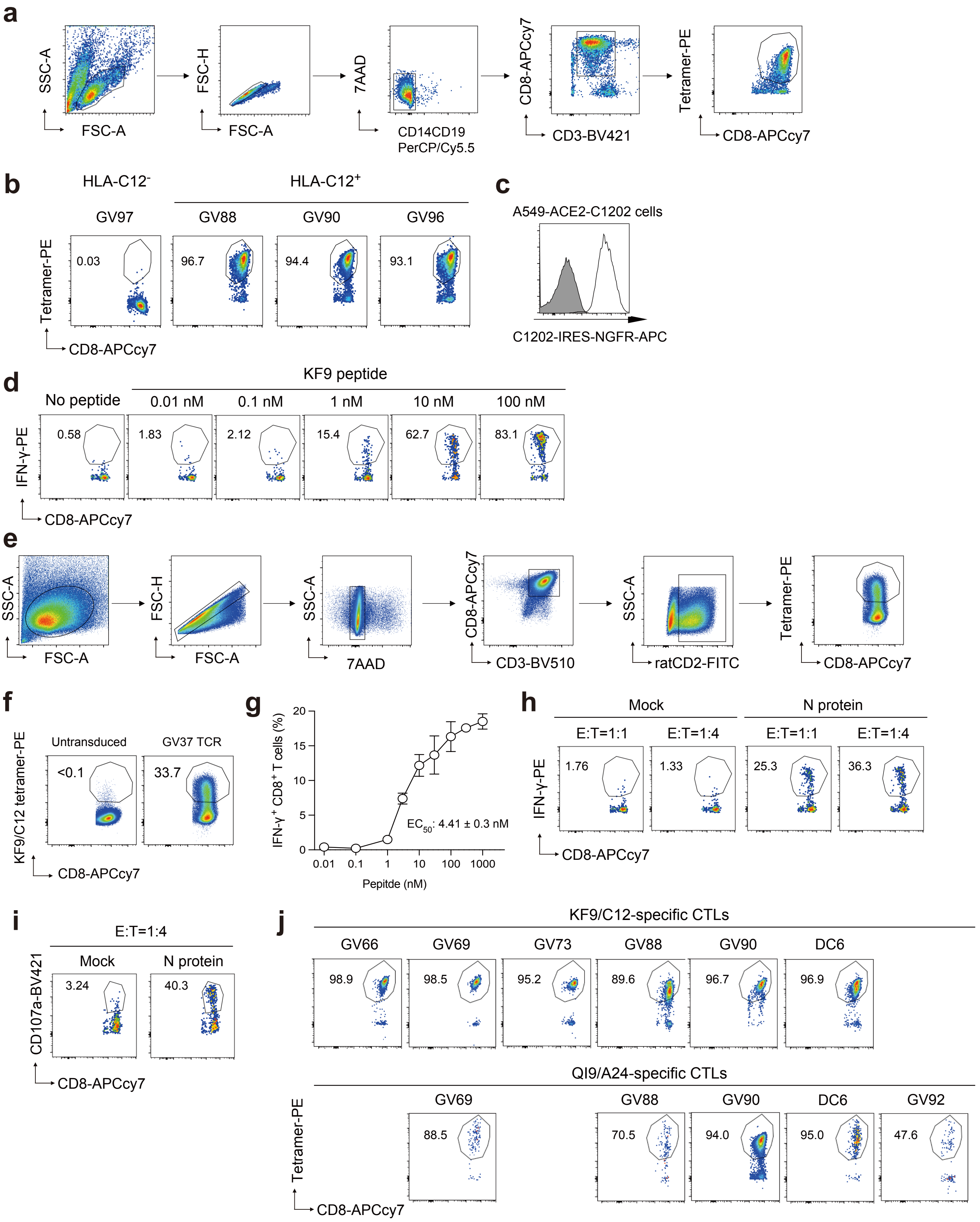

Supplementary Fig. 4 I Cell lines, gating strategy, cell lines, and peptides used in this study.
Flow cytometry gating strategy of KF9 stimulated-T cell lines from donor GV73 (**a**) and donors GV88, GV90 and GV96 (**b**). **c**, NGFR expression of A549-ACE2-C1202-IRES-NGFR cells. A549-ACE2 parental (shaded histogram) and FACS-sorted A549-ACE2-C1202-IRES-NGFR cells (open histogram) are shown. **d** Representative FACS plots of IFN-γ$^+$ activated KF9-specific T cell lines in peptide titration. **e-f** Flow cytometry gating strategy. (**e**) of GV37 TCR-transduced-CD8$^+$ T cells from a HLA-C*12:02- or HLA-C*14:02-negative donor and FACS plots of KC9/C12 tetramer$^+$ population of untransduced and GV37 TCR-transduced CD8$^+$ T cells (**f**). **g** The level of IFN-γ production of GV37 TCR-transduced CD8$^+$ T cells in stimulation with the KF9 peptide. **h-i** Representative FACS plots of IFN-γ$^+$ activated KF9-specific T cell lines in stimulation with A549-ACE2-C1202 cells expressing N protein (**h**) and CD107a$^+$ activated T cell lines (**i**) from donor GV88. **j** Representative FACS plots of tetramer$^+$ T cells lines specific for KF9/C12 and QI9/A24 used in **Fig. 5d** and **5e**.

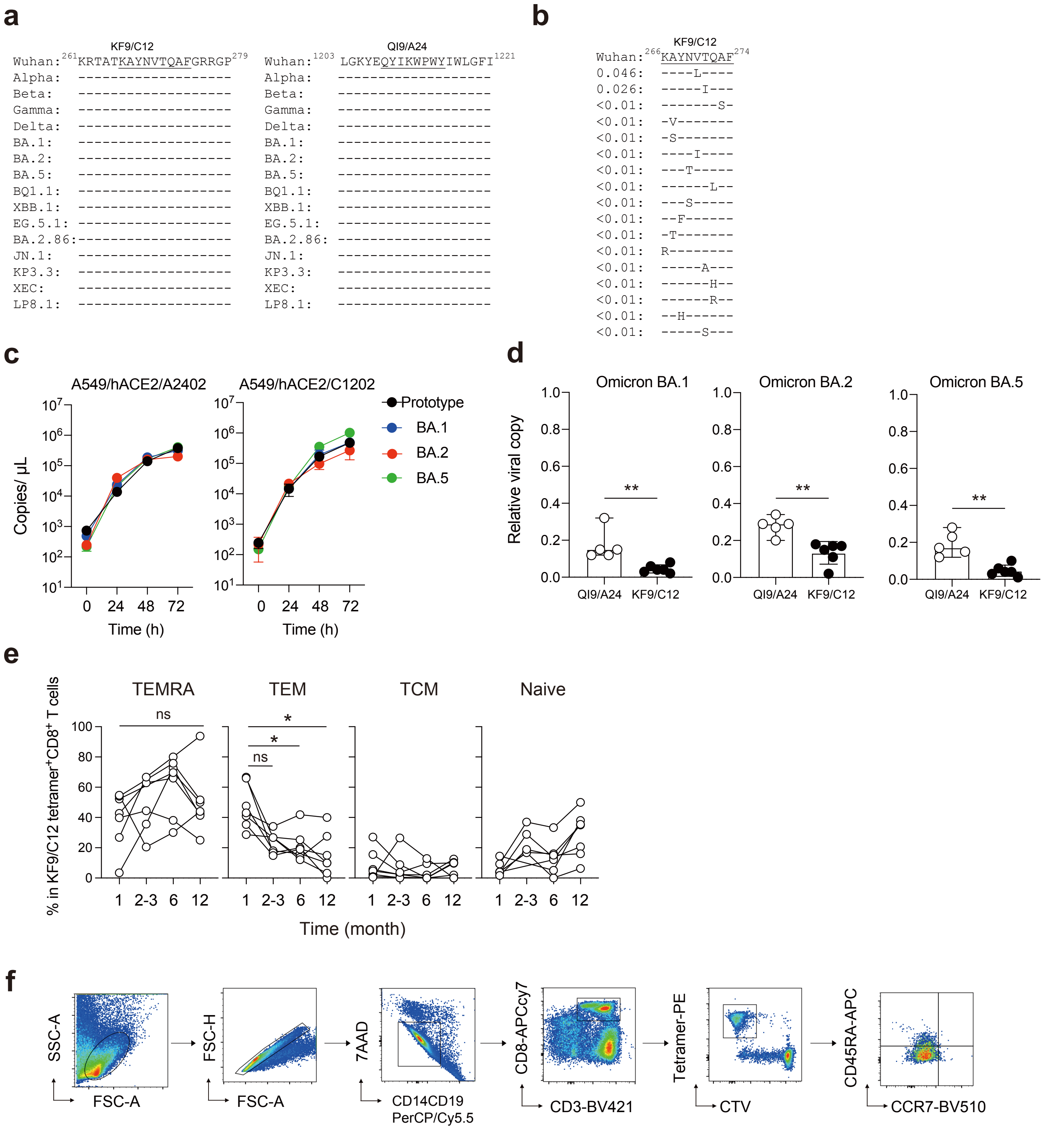

Supplementary Fig. 5 | Sequence alignment, viral replication and gating strategy used in this study. a Sequence alignment of KF9/C12 and QI9/A24 epitopes in various variants: SARS-CoV-2 Wuhan strain (DDBJ Accession ID: LC528232), Alpha (GISAID Accession ID: EPI_ISL_768526), Beta (GISAID Accession ID: EPI_ISL_1123289), Gamma (GISAID Accession ID: EPI_ISL_833366), Delta (GISAID Accession ID: EPI_ISL_2080609), Omicron/BA.1 (GISAID Accession ID: EPI_ISL_8559478), Omicron/BA.2 (GISAID Accession ID: EPI_ISL_9595859), Omicron/BA.5 (GISAID Accession ID: EPI_ISL_12812500), Omicron/BQ.1.1 (GISAID Accession ID: EPI_ISL_15579783), Omicron/XBB.1 (GISAID Accession ID: EPI_ISL_15669344), Omicron/EG.5.1 (GISAID Accession ID: EPI_ISL_18072016), Omicron/BA.2.86 (GISAID Accession ID: EPI_ISL_18110065), Omicron/JN.1 (GISAID Accession ID: EPI_ISL_18313756), Omicron/KP3.3 (GISAID Accession ID: EPI_ISL_19348437) , Omicron/XEC (GISAID Accession ID: EPI_ISL_19393351) and Omicron/LP8.1 (GISAID Accession ID: EPI_ISL_19715242). **b** Frequency of SARS-CoV-2 genomes harboring mutations within the KF9 epitope region in 5,378,593 SARS-CoV-2 genome sequences. The percentage is indicated on the left, and corresponding amino acid variants are shown on the right. Only variants occurring at a frequency of 10 or more are shown. **c** Viral replication of A549/hACE2/C1202 or A2402 cells infected with various virus strains. **d** Inhibition of viral replication of Omicron BA.1, BA.2 and BA.5 by KF9-specific T cells**. e** Percentage of TEMRA, TEM, TCM, and Naïve phenotypes in KF9/C12 tetramer$^+$ populations 1, 2-3, 6 and 12 months after infection. **f** Flow cytometry gating strategy of proliferating KF9/C12-specific T cells in **Fig. 6d and 6e**.

Supplementary Table 1. ***In silico*** **analysis of HLA binding using NetMHCpan4.1 and comparison with HLA-C*12:02, related to Figure 2f.**

**HLA allele**		**rank%[a]**	Similarity to HLA-C*12:02	Amino acid difference to HLA-C*12:02
HLA-A	A*02:01	7.799	88.30%	43
	A*02:06	2.274	88.30%	43
	A*24:02	0.843	88.30%	43
	A*31:01	3.888	88.60%	42
	A*33:03	8.229	88.60%	42
HLA-B	B*07:02	0.898	92.90%	26
	B*15:01	0.052	93.20%	25
	B*44:03	2.263	89.30%	39
	B*46:01	0.004	95.10%	18
	B*51:01	1.170	91.00%	33
	B*52:01	0.042	90.70%	34
	B*54:01	4.100	93.20%	25
	B*58:01	0.077	91.30%	32
	B*59:01	0.909	91.00%	33
	B*67:01:01	0.994	93.20%	25
HLA-C	C*01:02	0.723	98.40%	6
	C*03:02	0.003	98.20%	7
	C*03:03	0.050	97.50%	9
	C*03:04	0.050	97.80%	8
	C*04:01	0.788	97.50%	9
	C*07:02	0.267	95.60%	16
	C*12:02	0.006	-	-
	C*14:02	0.414	98.60%	5
	C*14:03	0.413	98.40%	6

[a]Strong affinity scores (<0.5) are shown as red.
