# Supplementary material for: Molecular basis of potent antiviral HLA-C-restricted CD8+ T cell response to an immunodominant SARS-CoV-2 nucleocapsid epitope
Source: Nat Commun. 2025 Aug 28;16:8062. doi: 10.1038/s41467-025-63288-3 (PMC12394707; doi:10.1038/s41467-025-63288-3)
Supplement: Supplementary file 2 — Description of Additional Supplementary Files [file 41467_2025_63288_MOESM2_ESM.pdf]

Description of Additional Supplementary Files

Supplementary Data 1: TCR repertoire of KF9/C12-specific T cells.

Supplementary Data 2: Analysis of the TCR-peptide contacts in crystal structures of TCR-pMHC complexes.
