# Supplementary material for: Molecular basis of potent antiviral HLA-C-restricted CD8+ T cell response to an immunodominant SARS-CoV-2 nucleocapsid epitope
Source: Nat Commun. 2025 Aug 28;16:8062. doi: 10.1038/s41467-025-63288-3 (PMC12394707; doi:10.1038/s41467-025-63288-3)
Supplement: Supplementary file 5 — Reporting Summary [file 41467_2025_63288_MOESM5_ESM.pdf]

nature portfolio

Corresponding author(s): Chihiro Motozono

Last updated by author(s): Aug 5, 2025

Reporting Summary

Nature Portfolio wishes to improve the reproducibility of the work that we publish. This form provides structure for consistency and transparency in reporting. For further information on Nature Portfolio policies, see our Editorial Policies and the Editorial Policy Checklist.

Statistics

For all statistical analyses, confirm that the following items are present in the figure legend, table legend, main text, or Methods section.

n/a	Confirmed	
☐	☒	The exact sample size (*n*) for each experimental group/condition, given as a discrete number and unit of measurement
☐	☒	A statement on whether measurements were taken from distinct samples or whether the same sample was measured repeatedly
☐	☒	The statistical test(s) used AND whether they are one- or two-sided<br>*Only common tests should be described solely by name; describe more complex techniques in the Methods section.*
☒	☐	A description of all covariates tested
☐	☒	A description of any assumptions or corrections, such as tests of normality and adjustment for multiple comparisons
☐	☒	A full description of the statistical parameters including central tendency (e.g. means) or other basic estimates (e.g. regression coefficient) AND variation (e.g. standard deviation) or associated estimates of uncertainty (e.g. confidence intervals)
☐	☒	For null hypothesis testing, the test statistic (e.g. *F*, *t*, *r*) with confidence intervals, effect sizes, degrees of freedom and *P* value noted<br>*Give P values as exact values whenever suitable.*
☒	☐	For Bayesian analysis, information on the choice of priors and Markov chain Monte Carlo settings
☒	☐	For hierarchical and complex designs, identification of the appropriate level for tests and full reporting of outcomes
☒	☐	Estimates of effect sizes (e.g. Cohen's *d*, Pearson's *r*), indicating how they were calculated

Our web collection on statistics for biologists contains articles on many of the points above.

Software and code

Policy information about availability of computer code

Data collection	Flow cytometry data were collected by CytExpert2.6, BD FACSDiva v9.0 and SpectroFlow3.2.1. Virus replication data were collected by LightCycler® 96 System (Roche). TCR sensitivity assay data were collected by a CentroXS3 plate reader (Berthhold Technologies). ELIPOT data was collected by ImmunoSot (Cellular Technology Limited). Thermal stability was measured using the ViiA 7 Real-Time PCR machine (Thermofisher).The structural data was processed using XDS (https://xds.mr.mpg.de/) and CCP4 (https://www.ccp4.ac.uk/).
Data analysis	Flow cytmetry data was analyzed by Flojo v10. Statistical analysis was performed in Prism software (v10). The sequence data were analyzed by GENETYX v12 (GENETYX Corporation). ELIPOT data was analyzed by ImmunoSot 7.0.34.0 Professoional Analyzer DC. Surface plasmon resonance (SPR) data was analyzed by BIAevaluation (v3.1). All molecular graphics representations were created using PyMOL (version 1.20; copyright, Schrodinger,LLC). The TCR model was build using AlphaFold2 (https://alphafold.ebi.ac.uk/), COOT (https://www2.mrc-lmb.cam.ac.uk/personal/pemsley/coot/), PHENIX (https://phenix-online.org/) and PHASER (https://www.ccp4.ac.uk/).

For manuscripts utilizing custom algorithms or software that are central to the research but not yet described in published literature, software must be made available to editors and reviewers. We strongly encourage code deposition in a community repository (e.g. GitHub). See the Nature Portfolio guidelines for submitting code & software for further information.

Data

Policy information about availability of data

All manuscripts must include a data availability statement. This statement should provide the following information, where applicable:

- Accession codes, unique identifiers, or web links for publicly available datasets
- A description of any restrictions on data availability
- For clinical datasets or third party data, please ensure that the statement adheres to our policy

All the raw data are available upon the request. All databases and datasets used in this study are available from GISAID (https://www.gisaid.org), DDBJ (https://www.ddbj.nig.ac.jp/index.html), IMGT (https://www.imgt.org/IMGT_vquest/vquest), and NetMHC4.1pan (https://services.healthtech.dtu.dk/services/NetMHCpan-4.1/). The crystal structures are availbale on the PDB (https://www.rcsb.org/), with the PDB code 9F13 for the HLA-C*12:02-KF9 and the PDB code 9HLJ fro the GV37 TCR-HLA-C*12:02-KF9 structures.

Research involving human participants, their data, or biological material

Policy information about studies with human participants or human data. See also policy information about sex, gender (identity/presentation), and sexual orientation and race, ethnicity and racism.

Reporting on sex and gender	Age, sex and days after vaccination or infection are described in Table 1 and 2.
Reporting on race, ethnicity, or other socially relevant groupings	No reporting on race, ethnicity, or socially relevant groupings in reported in our data as it does not bare relevance to the nature of our study.
Population characteristics	Age, sex and days after vaccination or infection are described in Table 1 and 2.
Recruitment	Study participants voluntarily donated blood at Kumamoto University, Kyushu Medical Center, Kyushu University, Tokyo Metropolitan Cancer and Infectious Diseases Center Komagome Hospitalafter written informed consent was obtained. All donors were vaccinated with a mRNA vaccine (BNT162b2 or mRNA-1273) or COVID-19 convalescents.
Ethics oversight	All protocols involving human subjects recruited at Kumamoto University, Kyushu Medical Center, Kyushu University, Tokyo Metropolitan Cancer and Infectious Diseases Center Komagome Hospital were reviewed and approved by the Institutional Review Boards of Kumamoto University (approval numbers 2066 and 461). All human subjects provided written informed consent.

Note that full information on the approval of the study protocol must also be provided in the manuscript.

Field-specific reporting

Please select the one below that is the best fit for your research. If you are not sure, read the appropriate sections before making your selection.

☒ Life sciences ☐ Behavioural & social sciences ☐ Ecological, evolutionary & environmental sciences

For a reference copy of the document with all sections, see nature.com/documents/nr-reporting-summary-flat.pdf

Life sciences study design

All studies must disclose on these points even when the disclosure is negative.

Sample size	No sample size calculation was performed. Sample size was determined by the availability of samples.
Data exclusions	No data were excluded.
Replication	Experiments with human PBMCs could not be replicated due to limited PBMC numbers. However,Fig. 2b, c, e, f, Fig. 3c, d, Fig. 5a, b, c, d, e, Supplementary Fig. 1k, Supplementary Fig. 4g, and Supplementary Fig. 5c assays were performed in triplicate. Data are representative of two or three independent experiments. In Fig. 3c, representative blots of three independent experiments were shown. Experiments were successfully repeated at least twice on independent samples.
Randomization	Vaccinated and convalescent donors were not randomized since we include all volunteers and selected based on HLA-typing (HLA-C*12:02 positive or negative).
Blinding	Experiments were not blinded as all participants received the vaccine and the study is observational.

Reporting for specific materials, systems and methods

We require information from authors about some types of materials, experimental systems and methods used in many studies. Here, indicate whether each material, system or method listed is relevant to your study. If you are not sure if a list item applies to your research, read the appropriate section before selecting a response.

Materials & experimental systems

n/a	Involved in the study
☐	☒ Antibodies
☐	☒ Eukaryotic cell lines
☒	☐ Palaeontology and archaeology
☒	☐ Animals and other organisms
☐	☒ Clinical data
☒	☐ Dual use research of concern
☒	☐ Plants

Methods

n/a	Involved in the study
☒	☐ ChIP-seq
☐	☒ Flow cytometry
☒	☐ MRI-based neuroimaging

Antibodies

Antibodies used

ELISPOT:
Purified anti-human IFN-γ (Clone: 1-D1K, Mabtech, #3420-3-1000,1:500)
Biotinylated anti-human IFN-γ (Clone: 7-B6-1, Mabtech, #3420-6-250, 1:2000)

Flow cytometry:
FITC-labeled anti-human CD3 (Clone UCHT1, Biolegend, Cat# 300440, 1:100)
BV421-labeled anti-human CD3 (Clone UCHT1, Biolegend, Cat# 300434, 1:50)
APCcy7-labeled anti-human CD8 (Clone RPA-T8, Biolegend, Cat# 301016, 1:100)
PerCP/Cy5.5-labeled anti-human CD14 (Clone HCD14, Biolegend, Cat# 325622, 1:100)
PerCP/Cy5.5-labeled anti-human CD19 (Clone HIB19, Biolegend, Cat# 302230, 1:100)
BV510-labeled anti-human CCR7 (Clone G043H7, Biolegend, Cat# 300434, 1:25)
APC-labeled anti-human CD45RA (Clone HI100, Biolegend, Cat# 309809, 1:50)
APC-labeled anti-human CD137 (Clone 4B4-1, Biolegend, Cat# 309809, 1:50)
PEcy7-labeled anti-human CD25 (Clone M-A251, Biolegend, Cat# 356107, 1:50)
PE-labeled anti-human IFN-γ (Clone 4S.B3; BD Biosciences, Cat# 554552, 1:100)
BV421-labeled anti-human CD107a (Clone H4A3, Biolegend, Cat# 328626, 1:100)
APC-labeled anti-human CD271 (NGFR) (Clone ME20.4, Biolegend, Cat#345108, 1:50)
APC-labeled anti-human HLA-A, B, C (Clone w6/32, Biolegend, Cat# 311410, 1:50)
PE-labeled HLA class I-specific mAb (Clone TP25.99, Thermo Fisher Scientific, Cat#MA5-44116, 1:50)
FITC-labeled anti-ratCD2 (Clone OX-34, Biolegend, Cat# 201303, 1:100)
BV510-labeled anti-human CD3 (Clone OKT3, Biolegend, Cat# 317332, 1:50)

Validation

Antibodies were titrated in our laboratory prior to their use. All antibodies were titrated by us prior to use on patient PBMC samples from healthy donors to determine optimal staining concentrations, and activation markers assessed after PMA/ionomycin stimulation. Single color florescence controls are acquired for each antibody to ensure the percentage and mean florescence intensity of antibodies in use are working well.

Eukaryotic cell lines

Policy information about cell lines and Sex and Gender in Research

Cell line source(s)

The TCR-deficient jurkat cell and was provided by Dr. Hiroyuki Kishi. A549-human ACE2 cell was provided by Dr.Kei Sato. C1R-A2402 cell, B5201 and C1202 were provided by Dr. Masafumi Takiguchi.

Authentication

None of the cell lines were authenticated.

Mycoplasma contamination

All cell lines tested negative for mycoplasma contamination.

Commonly misidentified lines
(See ICLAC register)

None used.

Clinical data

Policy information about clinical studies
All manuscripts should comply with the ICMJE guidelines for publication of clinical research and a completed CONSORT checklist must be included with all submissions.

Clinical trial registration

N/A

Study protocol

N/A

Data collection

N/A

Outcomes | N/A

Plants

Seed stocks | N/A

Novel plant genotypes | N/A

Authentication | N/A

Flow Cytometry

Plots

Confirm that:

☒ The axis labels state the marker and fluorochrome used (e.g. CD4-FITC).

☒ The axis scales are clearly visible. Include numbers along axes only for bottom left plot of group (a 'group' is an analysis of identical markers).

☒ All plots are contour plots with outliers or pseudocolor plots.

☒ A numerical value for number of cells or percentage (with statistics) is provided.

Methodology

Sample preparation | Human PBMCs were obtained from thirty-one early pandemic convalescents (median age: 48, Range: 19–77, 74% male) (Table 1); twenty-four HLA-C*12:02+ convalescents (median age: 42.5, Range: 23-86, 58.3% male), thirty-eight HLA-C*12:02- convalescents (median age: 43.5, Range: 25-88, 63.2% male), five HLA-C*12:02+ seronegative (median age: 24, Range: 23-40, 80% male) and four HLA-C*12:02- seronegative donors (median age: 24, range: 20-24, 25% male) (Table 2). PBMCs were purified by a density gradient centrifugation and stored in liquid nitrogen until further use.

Instrument | BD FACS Cant II, Cytek Northern Lights and Cytoflex were used for acquisition of data and BD FACS Aria II for cell sorting.

Software | BD FACSDiva v9.0, SpectroFlow3.2.1, CytExpert and Flojo v10.

Cell population abundance | Only single cell sorting was performed, which was confirmed by the presence of TCR chains and reconstruction of TCR on the TCR-deficient jurkat cells.

Gating strategy | Gating strategy was shown in Supplementary Fig. 1, 4 and 5.

☒ Tick this box to confirm that a figure exemplifying the gating strategy is provided in the Supplementary Information.
